# Cost-effectiveness of 40-hour versus 100-hour vocational rehabilitation on work participation for workers on sick leave due to subacute or chronic musculoskeletal pain: study protocol for a randomized controlled trial

**DOI:** 10.1186/s13063-015-0861-4

**Published:** 2015-07-28

**Authors:** Timo T. Beemster, Judith M. van Velzen, Coen A.M. van Bennekom, Monique H.W. Frings-Dresen, Michiel F. Reneman

**Affiliations:** Department of Rehabilitation Medicine, Center for Rehabilitation, University of Groningen, University Medical Center Groningen, Groningen, The Netherlands; Department of Research and Development, Heliomare Rehabilitation Center, Wijk aan Zee, The Netherlands; Coronel Institute of Occupational Health, Academic Medical Center, University of Amsterdam, Amsterdam, The Netherlands

**Keywords:** vocational rehabilitation, subacute musculoskeletal pain, chronic musculoskeletal pain, work participation, cost-effectiveness, non-inferiority, randomized controlled trial, multicenter

## Abstract

**Background:**

Although vocational rehabilitation is a widely advocated intervention for workers on sick leave due to subacute or chronic nonspecific musculoskeletal pain, the optimal dosage of effective and cost-effective vocational rehabilitation remains unknown. The objective of this paper is to describe the design of a non-inferiority trial evaluating the effectiveness and cost-effectiveness of 40-h multidisciplinary vocational rehabilitation compared with 100-h multidisciplinary vocational rehabilitation on work participation for workers on sick leave due to subacute or chronic musculoskeletal pain.

**Methods/Design:**

A non-inferiority study design will be applied. The study population consists of workers who are on part-time or full-time sick leave due to subacute or chronic nonspecific musculoskeletal pain. Two multidisciplinary vocational rehabilitation programs following the bio-psychosocial approach will be evaluated in this study: 40-h vocational rehabilitation and 100-h vocational rehabilitation, both delivered over a maximum of 15 weeks. The 100-h vocational rehabilitation comprises five modules: work participation coordination, graded activity, cognitive behavioral therapy, group education, and relaxation. The 40-h vocational rehabilitation comprises work participation coordination and a well-reasoned choice from the other four modules. Four rehabilitation centers will participate in this study, each delivering both interventions. Patients will be randomized into one of the interventions, stratified for the duration of sick leave (<6 weeks or ≥6 weeks) and type of sick leave (part-time or full-time). The primary outcome is work participation, measured by self-reported sick leave days, and will be assessed at baseline, mid-term, discharge, and at 2, 4, 6, 8, 10, and 12 months follow-up. Secondary outcomes are work ability, disability, quality of life, and physical functioning and will be assessed at baseline, discharge, and at 6 and 12 months follow-up. Cost outcomes are absenteeism, presenteeism, healthcare usage, and travelling costs. Cost-effectiveness will be evaluated from the societal and employer perspectives.

**Discussion:**

The results obtained from this study will be useful for vocational rehabilitation practice and will provide stakeholders with relevant insights into two versions of vocational rehabilitation.

**Trial registration:**

Dutch Trial Register identifier: NTR4362 (registered 17 March 2014).

## Background

Chronic musculoskeletal pain is a major health problem associated with decreased functioning and quality of life, sick leave, and increased direct and indirect medical costs [1–4]. The majority of the costs (48 to 88 %) are attributed to indirect costs due to sick leave from work or productivity loss while at work [5, 6]. Chronic musculoskeletal pain arises when acute musculoskeletal pain does not disappear within 6 weeks, which occurs in 10–20 % of the cases [7]. After a duration of 6 weeks, it is considered subacute musculoskeletal pain (SMP), and if the pain is still present after 12 weeks, it is considered chronic musculoskeletal pain (CMP) [8]. If there is no clear medical explanation, the chronic musculoskeletal pain is called “nonspecific.”

Vocational rehabilitation is a widely advocated intervention for sick-listed workers with subacute or chronic nonspecific musculoskeletal pain [9–12]. Vocational rehabilitation is “a multiprofessional evidence-based approach that is provided in different settings, services, and activities to working-age individuals with health-related impairments, limitations, or restrictions with work functioning and whose primary aim is to optimize work participation” [13]. In addition, work participation is conceptualized as the involvement in work roles or the lived experience of work. Work participation restriction refers to problems an individual may experience at work. Examples include number of hours lost from work (that is, absenteeism), underperforming job expectations, reduced desired employment (for example, part-time employment, short-term disability, long-term disability, premature retirement, or fewer working hours than desired), and reduced career growth [14]. However, in this paper, work participation (restriction) is expressed as the number of sick leave days due to subacute or chronic musculoskeletal pain. Research shows that vocational rehabilitation improves return to work [9–12, 15–22], and thus facilitates work participation. However, the dose-effect relation of vocational rehabilitation on work participation is unclear. Several reviews on the effectiveness of vocational rehabilitation on work participation for sick-listed workers with SMP and CMP show a wide range in treatment hours [9, 11, 20, 23]. In addition, a systematic review revealed a range of 6.4 to 196.8 hours in pain rehabilitation programs [23]. So far, only one randomized controlled trial has compared the dose-effect relation of vocational rehabilitation (VR) [16]. Sick-listed workers with CMP were classified at baseline as good, medium, or poor based on their prognosis for return to work (that is, return to work defined by the authors as absence of sick pay or related benefits in a given month), and were thereafter randomized to extensive VR (approximately 120 treatment hours), light VR (approximately 20 to 30 treatment hours), or care as usual (referred back to general practitioner). After 14 months follow-up, the participants classified with poor prognosis benefited most from the extensive VR, resulting in higher return to work rates, whereas patients classified with medium prognosis benefited from both the light and extensive programs on improving return to work rates. In another paper, but using the same study construct and population as in the Haldorsen trial [16], results were obtained without the prognosis on return to work (that is, good, medium, or poor) and on a follow-up period of two years. After 2 years follow-up, the light VR resulted in the highest return to work rates compared with usual care, but significance was only found in men. Additionally, the authors found no significant difference on return to work rates between light and extensive VR or between extensive VR and usual care [24].

As resources in healthcare are scarce, it is necessary to provide stakeholders information on the cost-effectiveness of intervention programs. Economic evaluations (cost-effectiveness studies) provide information on the relative efficiency of two or more alternative interventions. The main aspects of any economic evaluation are to identify, measure, value, and compare the costs and consequences of alternatives [25]. A randomized controlled trial found that a participatory approach (approximately 40 treatment hours consisting of a workplace intervention and graded activity) for sick-listed patients with chronic back pain was cost-effective on work participation (that is, return to work) compared with usual care [18]. Similar interventions conducted in subacute low back pain patients also show promising results on cost-effectiveness [21, 24, 26, 27]. However, there are no studies known that compare the cost-effectiveness of two (or more) vocational rehabilitation programs. To provide relevant stakeholders (that is, patients, referrers, employers, vocational rehabilitation centers, healthcare insurers, and policy makers) with information about effective and cost-effective vocational rehabilitation, a comparison of two versions of vocational rehabilitation is needed.

### Objectives

The objective of this paper is to describe the design of a multicenter, randomized, non-inferiority study to evaluate the effectiveness and cost-effectiveness of 40-h vocational rehabilitation compared with 100-h vocational rehabilitation on work participation for patients with subacute or chronic musculoskeletal pain and with sick leave from work. We hypothesize that 40-h VR will be noninferior on work participation and cost-effective in comparison with 100-h VR.

The research questions are:

I) For workers on sick leave due to subacute or chronic musculoskeletal pain, is 40-h vocational rehabilitation noninferior on work participation compared with 100-h vocational rehabilitation?
II) For workers on sick leave due to subacute or chronic musculoskeletal pain, is 40-h vocational rehabilitation cost-effective compared with 100-h vocational rehabilitation?

## Methods/Design

### CONSORT

In the description of our study design, we follow the Consolidated Standards of Reporting Trials (CONSORT statement) with the extension of reporting on non-inferiority trials [28].

### Organization of the study

**Fig. 1:**
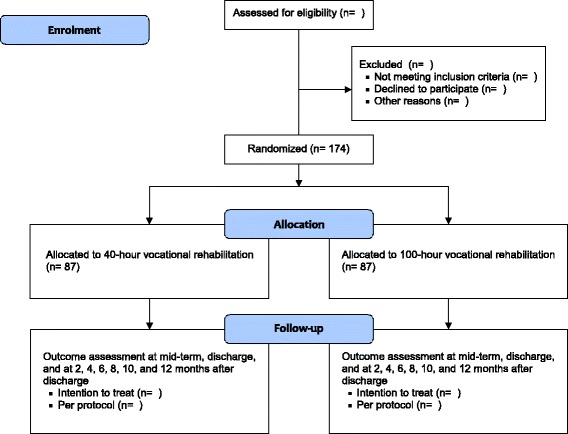
Flow chart of the design of the study

Approval for the study has been obtained by the Medical Ethics Committee of the Academic Medical Center, Amsterdam, the Netherlands (approval number: 2013_366). The trial is registered in the Dutch Trial Register (http://www.trialregister.nl/trialreg/index.asp) with identification number NTR4362. All participants will sign written informed consent forms and will be insured according to Dutch Law in case of any damage caused by participation in the study. Figure 1 shows a flow chart of the design of the study.

### Study design

A multicenter, randomized, 12-month follow-up, non-inferiority study design will be performed to evaluate the effectiveness and cost-effectiveness on work participation of 40-h versus 100-h vocational rehabilitation for patients with subacute or chronic musculoskeletal pain and on sick leave from work.

### Study population

The inclusion criteria for this study are as follows: 1) individuals of working age (18 to 65 years); 2) suffering from subacute (6 to 12 weeks) or chronic (>12 weeks) nonspecific musculoskeletal pain such as back, neck, shoulder, widespread pain, Whiplash Associated Disorder (WAD I or II), or fibromyalgia; 3) having paid work (employed or self-employed) for at least 12 hours per week; 4) the expectation that the employment or self-employment will not be terminated in the year following the vocational rehabilitation program; 5) having short-term (<6 weeks) or long-term (≥6 weeks) part-time or full-time sick leave; 6) being able to understand Dutch and able to complete questionnaires in Dutch; 7) having the motivation to participate in vocational rehabilitation aimed at optimizing work participation; 8) reimbursement of program costs by the employer (that is, the work participation coordination module, see Appendix 1); 9) having an email address; and 10) having granted informed consent. The exclusion criteria for this study is having comorbidities that are the primary reason for sick leave, such as acute or specific medical problems, clinical depression or burnout, severe asthmatic symptoms, diagnosed chronic fatigue, and neuropathy.

### Setting

Patients will be recruited between November 2014 and August 2016. The study will be performed in four vocational rehabilitation centers in the Netherlands that are part of a nationwide network of twelve VR centers. The four participating centers in this study are geographically spread across the Netherlands and have been selected according to the number of patients expected to be referred in 2014 to 2016.

### Recruitment of participants

Recruitment of participants occurs in five steps; the first three steps are regular steps and the last two steps have been added especially for this study. Step 1. Patients will be referred to one of the four participating centers by either an occupational physician, medical specialist, general practitioner, or employer. Step 2. A rehabilitation physician (RP) will assess the patient’s medical history, bio-psychosocial restrictions, and work-related limitations. Step 3. A multidisciplinary screening comprising a mental, physical and occupational assessment will take place, which will be performed by a psychologist, physiotherapist, and occupational specialist. Step 4. After completing the multidisciplinary screening, the patient will be provided with verbal and written information about the study. When all study criteria have been met, which will be decided by the RP, the patient will be asked to sign the informed consent form. Step 5. When the patient has granted written informed consent, the patient will be randomized into 40-h or 100-h vocational rehabilitation.

### Interventions

#### 40-hour vocational rehabilitation

The 40-h vocational rehabilitation is a multidisciplinary bio-psychosocial [29] group-based program and consists of work participation coordination (10 h), and a choice of 30 h of a set of modules offered in the 100-h vocational rehabilitation program, such as graded activity, cognitive behavioral therapy, group education, and relaxation. These modules are described in detail in Appendix 1. Since the choice of 30 h of modules will be prioritized by the multidisciplinary screening team after the multidisciplinary screening at baseline, the content may differ among patients. The 40-h VR lasts a maximum of 40 h in 15 weeks. Each rehabilitation center will prioritize the number of sessions per participant per week, but the following framework will be a guideline for the rehabilitation centers: weeks 1-5 two sessions/week, weeks 6-10 one session/week, weeks 11-15 2-3 sessions in five weeks. The 40-h VR program will be extended in case of the following: a patient’s percentage of working hours per week pertaining to contract hours at discharge compared with working hours per week pertaining to contract hours at baseline is extended by 25 to 50 %, and the multidisciplinary team expresses strong arguments that the patient is likely to benefit from the extension. However, this protocol deviation should occur in no more than 5 % of the cases. This percentage is arbitrarily chosen by the authors of this paper; if more than 5 % of the participants deviate from the study protocol, the robustness on non-inferiority (that is, research question 1) will decline [30].

#### 100-hour vocational rehabilitation

The 100-h vocational rehabilitation is a multidisciplinary bio-psychosocial group-based program and encompasses a set of modules: work participation coordination, graded activity, cognitive behavioral therapy, group education, and relaxation. These modules are described in detail in Appendix 1. The 100-h VR consists of approximately 100 h and is an existing VR program in the Netherlands conducted by 12 rehabilitation centers, four of which will participate in this study. The 100-h VR is delivered over a period of 15 weeks with two sessions (approximately 3.5 h/session) per week. The 100-h VR appears similar to other VR trials [17, 31] but has a longer duration (in weeks) and consists of more graded activity hours compared to similar studies [18, 21, 32].

### Data collection

**Fig. 2:**
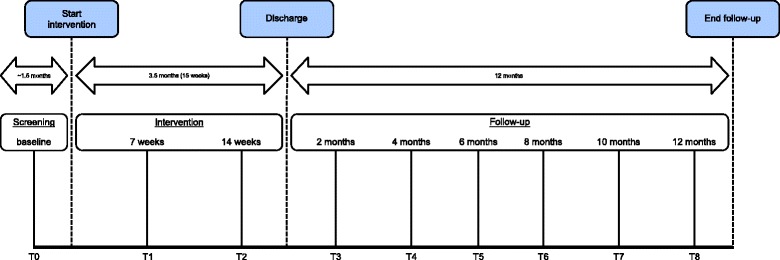
Timing of data collection

Self-reported data will be collected using web-based questionnaires. Data will be collected at baseline (that is, before and during the multidisciplinary screening, T0), 7 weeks after the start of the intervention (midterm, T1), 14 weeks after the start of the intervention (discharge, T2), and at 2, 4, 6, 8, 10 and 12 months follow-up after discharge (T3-T8). Figure 2 shows the timing of the data collection. In addition, pilot data show an expected delay of approximately 1.5 months between baseline and the start of the intervention. At each data point, participants will receive an email with login data and the request to complete questionnaires on a website. If participants do not complete the questionnaire within a week, they will receive a reminder email. If the questionnaire is not completed after this reminder, patients will be telephoned by a researcher (TB), who will ask patients to complete the questionnaire. Table 1 presents the outcome measures of the data collection.

**Table 1:** Outcome measures for each of the measurement moments

Outcomes	Time measured			Follow-up					
	Baseline	Midterm	Discharge	2 months	4 months	6 months	8 months	10 months	12 months
	T0	T1	T2	T3	T4	T5	T6	T7	T8
Descriptive variables									
Demographic variables	x								
General perceived health	x								
Work-related psychosocial factors	x								
Self-efficacy of work participation	x								
Pain intensity	x		x						
Fatigue	x								
Work tolerance functions	x								
Outcome measures									
Primary									
Work participation^a^	x	x	x	x	x	x	x	x	x
Secondary									
Work ability	x		x			x			x
Disability	x		x			x			x
Physical functioning	x		x			x			x
Quality of life	x		x			x			x
Costs^a^									
Presenteeism	x	x	x	x	x	x	x	x	x
Health care usage	x		x		x		x		x

^a^The primary outcome *work participation* will also serve as a cost outcome (that is, absenteeism)

### Outcome measures

The selection process of the questionnaires used in this study and information about their validity and reliability is described in a core set paper [33]. Primary, secondary, and cost outcomes will be assessed to answer the research questions.

#### Primary outcome

The primary outcome in this study is work participation expressed as total sick leave days due to subacute or chronic musculoskeletal pain. Total sick leave days will be calculated from the start of the intervention until 12 months follow-up after discharge. Sick leave will be measured using the absenteeism items of the iMTA (institute for Medical Technology Assessment) Productivity Cost Questionnaire (iPCQ) [34]. The questionnaire has a recall period of 4 weeks and measures sick leave on working days and on a generic basis (that is, the reason for sick leave is not asked). We have made slight adaptations to measure sick leave specifically (that is, related to subacute or chronic musculoskeletal pain, or other reasons such as flu), and we have added an item to assess the working hours at this moment: “Are you working for the full number of hours you were contracted for?” This question has three possible answers: “yes,” “no, I am partly at work,” and “no, I am on 100 % sick leave.” After the answer “no, I am partly at work” the participant is asked to fill in the number of hours he/she is working per week at that moment. The iPCQ is the result of combining two existing Dutch questionnaires (PRODISQ and SF-HLQ), and is recommended by the Dutch guideline for health economic evaluations [35]. The iPCQ has been translated by a professional language institution into a patient-friendly version using more simple language, thereby increasing the feasibility and validity of the questionnaire [34]. A population with mental health problems showed a satisfactory reliability regarding the iPCQ absenteeism items (icc 0.83) [36]. Until now, reliability of the iPCQ has not been tested in our study population. This needs to be done in the near future.

#### Secondary outcomes

Secondary outcomes include the following:

1. Work ability will be measured using a single item of the Work Ability Index (WAI) [37]. The current work ability compared to lifetime best work ability can be scored on a 0 to 10 response scale, where 0 represents “completely unable to work” and 10 represents “work ability at its best”.
2. Disability will be measured using the Pain Disability Index (PDI). The PDI is a 7-item questionnaire for investigating the magnitude of the self-reported pain-related disability, independent of region of pain or pain-related diagnosis. The PDI measures family/home responsibilities, recreation, social activity, occupation, sexual behavior, self-care, and life support activity. The questionnaire is constructed according to a 0 to 10 numeric rating scale in which 0 means no disability and 10 maximum disability. Total scores can range from 0 to 70, with higher scores reflecting higher interference of pain with daily activities [38, 39].
3. Physical functioning will be measured using the physical functioning subscale of the RAND-36. The questionnaire assesses self-reported physical functioning independent of (pain) diagnosis [40]. The physical functioning scale consists of 10 questions with three possible answers: “yes, limited a lot,” “yes, limited a little,” and “no, not limited at all.” The total score can range from 0 to 100, with higher scores indicating better physical functioning. The validity and reliability of the Dutch version are good [41].
4. Quality of life will be measured using the validated Dutch version of the EuroQol-5D (EQ-5D) [42, 43]. The EQ-5D measures five dimensions: mobility, self-care, activities of daily life, pain and anxiety/depression on a categorical scale (1 to 3). The EQ-5D is a widely employed instrument used to assess health-related quality of life (QoL), and is recommended by the Dutch guideline for health economic evaluations [35]. To allow comparison between several conditions and interventions, Quality Adjusted Life Years (QALYs) will be calculated in three steps. First, the EQ-5D scores (measured at baseline, discharge, 6-month follow-up, and 12-month follow-up) will be converted to utility scores using the Dutch EQ-5D tariff [43]. Second, QALYs will be calculated from three time periods (1 = baseline to discharge, 2 = discharge to the 6-month follow-up, 3 = 6-month follow-up to the 12-month follow-up). Third, one summated QALY will be calculated from the calculated QALYs in step two.

#### Cost outcomes

The following outcomes will be assessed to evaluate the cost-effectiveness of the 40-h VR compared with the 100-h VR:

1. Absenteeism data will be derived from the work participation (that is, primary outcome) data in this study.
2. Presenteeism will be assessed using the presenteeism items of the iPCQ [34]. The questionnaire measures the total days of mental or physical complaints at work, with a recall period of 4 weeks. The amount of work performed accompanied by mental or physical complaints is measured on a 0 to 10 response scale, where 0 represents “I couldn’t do anything,” to 5 “I could do about half of normal,” to 10 “I could do the same as normal.” A population with mental health problems showed good feasibility and validity [34], and moderate reliability for the number of days while impeded by mental or physical complaints (icc 0.56), and a satisfactory reliability for the efficiency rate (0 to 10) item (icc 0.73) [36].
3. Healthcare usage will be assessed using the Trimbos iMTA questionnaire for measuring Costs of Psychiatric Illnesses (TiC-P), module 1 [36, 44]. A recall period of 4 weeks is used in this questionnaire. Visits and consultations of the following healthcare providers were measured: general practitioner, physiotherapist, manual therapist, exercise therapist, occupational therapist, psychologist, insurance physician, medical specialists in hospitals, hospitalization (number of days), occupational physician, social worker, and dietician. Additional items were alternative care, home care, medication use, and job-related care like job coaches, ergonomic changes at the work site and reintegration specialists. Slight adaptations in the context and scope of health care practitioners were made to better match TiC-P to the target population (that is, from psychiatry to pain and work). Another modification was that visits and consultations were measured in both generic and sickness-specific terms. Research shows that health care usage assessment by means of self-reported questionnaires is reliable [45]. A population with mental health problems showed good feasibility, promising construct validity, good agreement on medical resource use (yes/no), and sufficient test-retest reliability on the number of contacts with the health care providers [36]. Until now, reliability of the TiC-P has not been tested in our study population. This needs to be done in the near future.

#### Patient characteristics

Patient characteristics will be collected at baseline (that is, before and during the multidisciplinary screening) to evaluate if randomization resulted in two prognostically comparable groups. The following characteristics will be collected:

1. Demographic variables: age, gender, marital status, nationality, body mass index (obtained from self-reported weight and height), educational level, and health condition [46, 47].
2. General perceived health will be assessed using a single item of the RAND-36 questionnaire [41].
3. Work-related psychosocial factors will be assessed using the Work Reintegration Questionnaire (WRQ). The questionnaire consists of 78 items distributed across eight scales: distress, illness behavior/coping, job strain, job satisfaction, job control, avoidance, perfectionism, and stressful home situation. The questionnaire was developed and validated in Dutch (VAR: vragenlijst arbeidsreintegratie) [48, 49].
4. Self-efficacy of work participation will be assessed on a 0 to 10 response scale. Participants rate the certainty that they will be working in 6 month’s time, where 0 represents “not at all certain” to 10 “extremely certain.” A score of ≥5 is associated with successful work participation after 6 months for workers with subacute back pain [50].
5. Pain intensity and fatigue will each be measured using two questions from an 11-point Numeric Rating Scale, ranging from 0 “no pain/fatigue” to 10 “worst possible pain/fatigue,” requiring patients to rate their worst and average intensity of the last 7 days [51].
6. Work tolerance functions will be assessed at baseline during the multidisciplinary screening using standardized lifting capacity tests from the Functional Capacity Evaluation (FCE) test battery: lifting low and/or overhead lifting. The lifting tests to be assessed depend on the individual’s work tasks. Procedures are described in detail elsewhere [52]. Lifting tests were found to be predictive of work participation in patients with musculoskeletal disorders [53].

### Non-inferiority hypothesis

A reduction in sick leave days of more than 30 days per year is deemed a clinically significant improvement on work participation [15, 17–19, 24]. A difference in sick leave days of 30 or fewer (from the start of the intervention until 12-months follow-up) between 40-h and 100-h VR is assigned as the margin of non-inferiority in this study. Our hypotheses are as follows:

- H0: μ1 - μ2 ≥30
- H1: μ1 - μ2 <30

H0 is the null hypothesis, and H1 is the alternative hypothesis, μ1 is the mean number of sick leave days in the 40-h VR group, and μ2 is the mean number of sick leave days in the 100-h VR group.

Non-inferiority is claimed if the upper bound of the one-sided 95 % confidence interval of the treatment effect difference (μ1 - μ2) on work participation does not exceed 30, which means that the risk of it being inferior is within acceptable boundaries [30]. We expect a normal distribution of the primary outcome work participation. If the data on the primary outcome does not follow a normal distribution, we will perform log transformations. The margin of non-inferiority will then be interpreted as a 28 % increase in the sick leave days’ difference of μ1 - μ2. We calculated this percentage as follows:

$$ 30/107 = 28\ \% $$

where 30 denotes the margin of non-inferiority and 107 the expected mean days of sick leave in the 100-h VR arm [15, 17–19, 24] during the timing of the data collection, which equals approximately 15.5 months (intervention period of 3.5 months + the follow-up period of 12 months, see Fig. 2).

### Statistical methods

All statistical analyses will be performed at the patient level, with descriptive statistics being used to compare the baseline measurements of the two intervention groups. If necessary, analyses will be adjusted for baseline differences. All analysis will be performed according to the intension-to-treat principle and the per protocol principle [30, 54]. To claim non-inferiority, both intention-to-treat and per protocol analysis must show non-inferiority [54]. Missing data on costs and effects will be assessed using multiple imputation techniques [25]. The imputation technique will depend on the results (that is, missing completely at random, missing at random, or missing not at random).

#### Effectiveness

The primary outcome work participation will be analyzed in three steps. Step 1. For every time point (that is, T0 - T8, see Fig. 2), we will present the number of sick leave days as an absolute number and as a percentage related to contract hours/month, in which the absolute number and percentages between a given time point and the preceding time point will be calculated using linear extrapolation, as recommended [36, 44, 55]. Step 2. We will calculate and present the cumulative total days of sick leave per month from the start of the intervention until the 12-month follow-up using an area under the curve for all measurement points, in which the number of sick leave days between a given time point and the preceding time point will be calculated using linear extrapolation. Step 3. Linear mixed models with multilevel analyses will be performed to assess non-inferiority between the two groups at the 12-month follow-up (that is, intervention period and 12-month follow-up) by means of 95 % confidence intervals (that is, the CI approach). To improve generalizability and comparability of this study with other studies, we will repeat step 3 at the following time intervals: I) discharge to 12-month follow-up; II) start of intervention to 6-month follow-up; III) discharge to 6-month follow-up. These additional analyses will contain no conclusions about non-inferiority and will be analyzed in the “classical” superiority manner.

A t-test or Mann–Whitney U test (in the case of no normal distribution) will be used to examine differences at discharge, at 6-month follow-up, and at 12-month follow-up (defined as the difference in outcome between baseline and last follow-up) in all secondary outcomes between the intervention groups. We will perform these analyses on superiority, thus without margins of noninferiority, as this is only relevant for the analysis of the primary outcome.

#### Cost-effectiveness

Various kinds of economic evaluations are recommended for the same study to inform all relevant stakeholders [25]. We will perform three types of cost analysis: cost-effectiveness analysis, cost-utility analysis, and cost-benefit analysis.

##### Cost-effectiveness analysis: societal perspective

The cost-effectiveness analysis (CEA) in this study will be evaluated from the societal perspective (that is, all costs related to the intervention will be taken into account irrespective of who pays for them). Costs consist of direct medical costs (that is, intervention costs, health care usage and travelling costs) and indirect costs (that is, productivity loss in paid work due to absenteeism and presenteeism). All costs will be summated for each individual patient. All summated costs will be indexed in euros for the reference year 2015. We will follow the friction cost method with a friction cost period of 160 days and an elasticity of 0.8 for the calculation of absenteeism costs [56], as recommended by the Dutch guideline for health economic evaluations [35] and described in detail elsewhere [34, 56]. To calculate the presenteeism costs, the costs of productivity losses will be multiplied by the number of workdays lost, with age and gender-specific productivity levels per paid employee indexed for the year 2015 [34, 35].

Both the incremental costs and incremental effects will be used to calculate the incremental cost-effectiveness ratio (ICER). The ICER will be calculated as (C1 − C0)/(E1 − E0), where C denotes the average per-participant costs and E denotes the effect on work participation in the 40-h and 100-h VR groups (subscripted 1 and 0). As absenteeism data will be used for the assessment of the effect ratio of the ICER, it will be excluded from the cost ratio part of the ICER. The ICER can be interpreted as the net costs (or savings) per extra unit of effect. In our study, the extra unit of effect equals a 1-day increase in work participation. To estimate uncertainty in the cost and effect data, nonparametric bootstraps will be used to simulate 5,000 ICERs [57]. To show statistical uncertainty on the results of cost-effectiveness, each simulated ICER will be plotted on a cost-effectiveness plane [58]. Although cost-effectiveness planes give a good impression of the uncertainty surrounding the ICER, they do not provide a summary measure of the joint uncertainty of costs and effects [25]. We will therefore perform cost-effectiveness acceptability curves (CEAC), which will provide insight into the probability that 40-h VR is cost-effective in comparison with 100-h VR [25].

##### Cost-utility analysis

A cost-utility analysis (CUA) will be conducted in which the incremental costs per QALY will be estimated and which will be presented on a cost-effectiveness plane and CEAC. Public policymakers may be interested in CUA because they can compare the results between several conditions and interventions [25].

##### Cost-benefit analysis: employer’s perspective

As employers reimburse the work participation coordination module in both the 40-h VR and 100-h VR, analysis from the employer’s perspective (that is, only the costs relevant to the employer will be considered, including intervention, absenteeism, and presenteeism costs) is useful. It is recommended to conduct cost-benefit analysis (CBA), in which both costs and consequences are measured in monetary units. In accordance with van Dongen et al. [25], we will perform a return on investment (ROI) analyses, in which three ROI metrics are calculated: (1) net benefits (NB), (2) benefit-cost ratio (BCR), and (3) ROI.

NB = benefits − costs

BCR = benefits/costs

ROI = (benefits − costs)/costs [*100]

Costs will be defined as intervention costs. Benefits will be defined as the difference in monetized outcome measures (that is, absenteeism and presenteeism costs) between 100-h and 40-h VR during the measurement period (that is, intervention period and follow-up, see Fig. 2), with positive benefits indicating reduced spending in the 40-h group. To estimate uncertainty, 95 % CIs around the benefit estimates and NB will be estimated by means of bootstrap confidence intervals. Financial returns of 40-h VR are positive if the following criteria are met: NB > 0, BCR > 1, and ROI > 0 % [25].

#### Sensitivity analyses

To assess the robustness of the results on cost-effectiveness, we will perform four sensitivity analyses. First, analyses will be performed using the complete cases only. Second, analyses will be performed in which the lost productivity costs will be calculated according to the human capital approach. In the human capital approach, total sick leave days are not fixed as in the friction cost approach, and elasticity is not required [25]. Third, analyses will be performed with sick leave and healthcare usage data that are related to subacute or chronic musculoskeletal pain. Fourth, the observed outliers with very high lost productivity will be excluded from the analysis.

### Sample size

A sample size of 174 is calculated to be sufficient (with a one-sided 95 % CI, 80 % power, alpha of 0.025, standard deviation of 80 and a margin of non-inferiority of 30 days) to establish non-inferiority of 40-h VR. The sample size calculation allowed for 15 % loss to follow-up - 10 % expected from comparable studies [18, 59, 60] and 5 % expected due to the extension of the program in the 40-h VR group. An intraclass correlation coefficient (ICC) of 0.05 is accounted for by the use of four rehabilitation centers with two clusters (40-h and 100-h VR) at each center [61, 62]. Because of the difference in program hours between 40-h and 100-h VR, we expect 40-h VR to benefit by 8 extra working days available during the intervention period. We accounted for this in the power calculation by using minus 8 as the expected mean difference between 40-h and 100-h VR. In our power calculation, we assumed a normal distribution of the primary outcome work participation. If the data on the primary outcome does not follow a normal distribution, we will perform log transformations. As previously stated, we will allow 28 % as the margin of non-inferiority when the data is log transformed.

According to the number of patients expected to be referred to the four participating rehabilitation centers per year (approximately 350), and after accounting for two-thirds of non-participation in the study according to Lasagna Law [63], we expect an inclusion of 115 participants per year for this study. Hence, our inclusion period will cover approximately 18 months, and the data collection period will cover 2 years and 9 months.

### Randomization

An independent statistician prepared the randomization by using computer-generated randomization tables. To prevent unequal randomization, employees are pre-stratified by duration of sick leave (short-term <6 weeks or long-term ≥6 weeks) and whether they are on full-time (100 %) or part-time (≤99 %) sick leave. Block randomization with blocks of four will be applied to ensure equal group sizes within each stratum. A separate block randomization table is generated for each of the four participating vocational rehabilitation centers. For each stratum, the researcher will prepare opaque, sequentially numbered, and sealed coded envelopes, with a note for either the 40-h VR or 100-h VR. After the multidisciplinary screening (at baseline), the multidisciplinary screening team and rehabilitation physician will fulfil all study criteria. If participants meet all criteria, they will be allocated to 40-h or 100-h VR. Treatment allocation will be performed by a member of the multidisciplinary screening team (MST) at each center and can be performed at the center or via telephone (this will differ among the centers). The MST member hands over two envelopes (left over) of that stratum, and the patient is asked to pick one of the envelopes, open the envelope and sign the note. In the case of telephone allocation, the MST member will ask the patient to sign informed consent and to return it via a reply envelope. When the signed informed consent is received, the MST member will perform the treatment allocation without the patient. After randomization, a research assistant will make an appointment for the patient’s first intervention date.

### Blinding

Blinding in this study is not possible because of the nature of the intervention. However, the data analyst will remain blinded to the allocation. Participants will complete self-reported web-based questionnaires outside the study setting, so the multidisciplinary intervention team has no influence on the outcome assessment. After randomization, all participants are labelled with a research code consisting of a unique consecutive number. An independent researcher will maintain the coding scheme. Data analysis will be performed using this research code to guarantee that analyses of the data by the researcher will be blinded.

### Co-interventions and compliance

The patients’ self-reported healthcare usage data will be used for the assessment of co-interventions. Compliance will be assessed using information about attendance to the program and compliance to the treatment protocol and will be assessed after each intervention session in an electronic log by a member of the multidisciplinary intervention team (MIT). Furthermore, the MIT member will determine at discharge if the program was completed as planned. This will be assessed on a binary scale: “program completed as planned,” or “program deviated.” In the latter case, a closed question follows: program deviated due to “early discontinuation due to adverse events such as accident, surgery, or major private event,” “early discontinuation due to goals being achieved,” “extension of intervention program due to non-achievement of goals,” or “other reasons.” In the case of an early discontinuation or extension of the program, the number of deviated weeks will be reported. The information about compliance will be applied to perform the per protocol analyses.

## Discussion

The purpose of the presented study is to evaluate the effectiveness and cost-effectiveness of 40-h vocational rehabilitation versus 100-h vocational rehabilitation on work participation for sick-listed workers due to subacute or chronic nonspecific musculoskeletal pain. We hypothesize that there is non-inferiority on work participation after a 12-month follow-up period (including the intervention period and 12 months follow-up) between both programs, and we expect cost-effectiveness of 40-h VR in comparison with 100-h VR.

### Context of this study

In the Netherlands, both employer and employee are responsible for the work participation process of the sick-listed employee during the first 2 years of sick leave. The employer and employee can be supported by a certified reintegration company and/or an occupational physician (OP). In the first 2 years of sickness, the employer is responsible for the costs of wage replacement, which is regulated by the Dutch Gatekeeper Improvement Act [64]. As a result of this act, the employer has to reimburse the work participation coordination module (costs: approximately €1,200) for both interventions performed in this study. The other intervention modules are reimbursed by healthcare insurers.

### Methodological considerations

The first methodological consideration of this study is that we were not able to fulfil the recommended steps for the composition of a margin of non-inferiority [30, 65]. This was because there is currently no historical data, such as meta-analysis, comparing the 100-h vocational rehabilitation with usual care. However, our non-inferiority margin is based on results from five randomized controlled trials evaluating multidisciplinary vocational rehabilitation compared with control interventions (that is, usual care, such as occupational physician, physical therapist, occupational therapist, *etcetera*) [15, 17–19, 24]. These studies found 43 days as the mean difference ((41.9 + 53.7 + 42 + 60.5 + 17.5)/5) in days on sick leave after one year follow-up in favor of multidisciplinary vocational rehabilitation. Consequently, we have decided that 30 is an acceptable margin of non-inferiority. One can argue that this limit is too wide, and that claiming non-inferiority could be achieved too simply, but for claiming non-inferiority, the upper bound of the one-sided 95 % confidence interval of the treatment effect difference (40-h VR – 100-h VR) must be 30 or less. Furthermore, when the margin of non-inferiority of 30 is reached after 12 months follow-up, this is in fact 13 days (43–30) better than if a patient had been referred to usual care (occupational physician, physical therapist, occupational therapist, *etcetera*). A mean saving of 10 sick days per year is considered the smallest effect that would be clinically worthwhile [66]. We therefore consider 30 as a reasonable margin of non-inferiority. The second methodological consideration of this study is the slight differences between the participating rehabilitation centers. For instance, each center has its own logistic restrictions, such as a restriction in intervention facilities (that is, equipment, building); and centers have evolved their own methods over the years. This may lead to interpretation issues in analyzing the blended results of the four centers. However, we solved this problem by multilevel analyses and by performing both interventions at each center, that is, by randomizing at the participant level. Although performing both interventions at the center level may introduce contamination, we consider that the advantages of randomization at the participant level outweigh the disadvantages of contamination.

### Strengths and limitations of this study

The first strength of this study is the assessment of the primary outcome work participation with self-reported questionnaires with a recall period of one month. This recall period will prevent recall bias. In addition, self-reported data about work participation has been shown to be a reliable alternative compared with electronic databases [67]. The second strength of this study is the analysis of the cost-effectiveness from both the societal and the employer’s perspective. It is important to provide employers with information on the return on investment of both interventions, as this will help them to consider the right treatment. A third strength of this study is that we consider presenteeism in the cost-effectiveness evaluation. Although most cost-effectiveness studies do not assess presenteeism [17, 18, 31, 35, 68], it is meaningful to take into account since the costs related to it are enormous, as shown by Lötters et al. [69], who found that for workers who returned to work after musculoskeletal disorders, the median loss for an 8-h workday was 1.6 h, and this remained at 12-month follow-up. A final strength of this study is the participation of four rehabilitation centers, all working with the bio-psychosocial model as a blueprint. This will increase the generalizability of this study.

A limitation of this study is that it is not possible to blind the patients and the multidisciplinary intervention and screening team. This may result in non-compliance to the treatment protocol, because patients may be aware of which intervention parts they do not receive (especially in the 40-h VR group), whereas other patients in the same group will receive all intervention modules (see Appendix 1). When this deviation occurs on a large scale in the 40-h VR group, this will harm conclusions about non-inferiority. Another limitation of this study is that we do not correct for compensation costs, that is, when colleagues take over the work of the less productive employee in their regular working hours. This may overestimate presenteeism costs [70].

### Implications for practice

This study will provide essential knowledge about the dose-effect relation of vocational rehabilitation on work participation for workers on sick leave due to subacute or chronic musculoskeletal pain. The insights obtained from this study can be implemented in vocational rehabilitation practice, where centers would be able to judge which program (40-h or 100-h) fit their patient groups best. Moreover, if our hypothesis about the effectiveness and cost-effectiveness of 40-h VR compared with 100-h VR is valid, this will be beneficial for patients, employers, and health care insurers. Patients will benefit from a decline in intervention hours, which will result in more time for work participation and leisure. Employers will benefit from a higher return on their investments, and health care insurers will benefit from higher volumes of patients who can participate in vocational rehabilitation within the same amount of time and money or the same number of patients with lower costs.

## Trial status

Participant enrollment started in November 2014. Recruitment is expected to be completed by the end of August 2016, and the trial will conclude by the end of December 2017.

### Additional information

To place the results from the described cost-effectiveness study in perspective, the authors of this paper will also conduct a qualitative paper in which interviews with a random selection of the study population of the proposed RCT will be performed. The aim of these interviews will be to determine barriers and facilitators of the 40-h and 100-h VR on work participation. The authors will also conduct focus group interviews with the multidisciplinary intervention teams to explore their experiences with both programs.

## Appendix 1: Intervention modules

### Work participation coordination

Work participation coordination is carried out by a work participation coordinator [71] and encompasses a workplace visit, case management, and two evaluation moments. The workplace visit includes an ergonomic workplace analysis and a consultation with employer and employee (patient), with the aim of developing a work participation plan. Case management consists of individual coaching sessions with the patient and unplanned ad hoc conversations with the patient during the program. The coaching style of the work participation coordinator is mainly based on solution-focused coaching [72, 73] and on empowerment [74]. The two evaluation moments include a report on progression in work participation, which will be performed at mid-term and discharge.

Dosage: 10 hours in both programs.

### Graded activity

Graded activity is based on the protocol designed by Lindstrom [75, 76] and adjusted to the Dutch situation [8, 32, 77]. The graded activity program is carried out by a physical therapist. The purpose of graded activity is to restore occupational functioning and to facilitate work participation. During the program, the patient has an active role and the physical therapist acts as a coach and supervisor, using a hands-off approach [77]. Graded activity is a time-contingent approach with an increase in load and complexity of movements. To attain physical reconditioning, the graded activity protocol may be supplemented with endurance exercises.

Dosage: 60 hours (2 × 2 hours per week) for patients in the 100-h VR group. The amount of graded activity in the 40-h VR group will differ per patient.

### Cognitive behavioral therapy

Cognitive behavioral therapy (CBT) is carried out by a psychologist and consists of individual sessions, group education, and unplanned ad hoc conversations during the program. The CBT sessions are based on solution-focused coaching [72, 73] and empowerment [74]. It encompasses items such as coping, cognition, communication, and self-control.

Dosage: the dosage differs per patient, but we factor in approximately 30 minutes per week for patients in the 100-h VR group, resulting in a total of 7.5 h. The amount of CBT in the 40-h VR group will differ per patient.

### Group education

Group education encompasses physical and mental topics, and will be carried out by a physical therapist and a psychologist. Physical topics are the effect of physical activity on the body (that is, training principles), chronic and acute pain, pain sensitization, anatomy and ergonomics, and pre- and post-exercise nutritional recommendations. Mental topics are empowerment, setting graded tasks, cognitive behavioral therapy, and coping with pain.

Dosage: 15 h (60 minutes per week) for patients in the 100-h VR group. The amount of group education in the 40-h VR group will differ per patient.

### Relaxation

Relaxation sessions are carried out by a physical therapist. Different techniques are employed, such as meditation, visualization, autogenic training, mindfulness, breath control, progressive relaxation, and reciprocal inhibition. The aim of relaxation is improved body awareness and experiencing the difference between tension and relaxation of the muscles.

Dosage: 7.5 h (30 minutes per week) for patients in the 100-h VR group. The amount of relaxation in the 40-h VR group will differ per patient.

## Abbreviations

BCR: benefit-cost ratio
CBA: cost-benefit analysis
CBT: cognitive behavioral therapy
CEA: cost-effectiveness analysis
CEAC: cost-effectiveness acceptability curves
CI: confidence interval
CMP: chronic musculoskeletal pain
CUA: cost-utility analysis
EQ-5D: EuroQol-5D
FCE: Functional Capacity Evaluation
ICC: intraclass correlation coefficient
ICER: incremental cost-effectiveness ratio
iPCQ: iMTA (institute for Medical Technology Assessment) Productivity Cost Questionnaire
MIT: multidisciplinary intervention team
MST: multidisciplinary screening team
NB: net benefits
OP: occupational physician
PDI: Pain Disability Index
QALYs: quality adjusted life years
QoL: quality of life
ROI: return on investment
RP: rehabilitation physician
RTW: return to work
SMP: subacute musculoskeletal pain
TiC-P: Trimbos iMTA Questionnaire for Measuring Costs of Psychiatric Illnesses
WAD: whiplash-associated disorder
WAI: Work Ability Index
WRQ: Work Reintegration Questionnaire
